# Supplementary material for: Comparison of lamina cribrosa properties and the peripapillary vessel density between branch retinal vein occlusion and normal-tension glaucoma
Source: PLoS One. 2020 Oct 2;15(10):e0240109. doi: 10.1371/journal.pone.0240109 (PMC7531791; doi:10.1371/journal.pone.0240109)
Supplement: Attachment — Submitted filename: response to Reviewers.docx [file pone.0240109.s001.docx]

Dear reviewers & editor

First of all, we would like to thank the reviewers for their time and valuable comments on our manuscript. We have addressed our opinions on each comment of the two reviewers in this response letter, and made several changes and corrections to our original manuscript.
We tried to revise our manuscript according to reviewers’ suggestions as much as possible, and we hope that the revisions in the manuscript and our accompanying responses will be sufficient to make our manuscript suitable for publication in PLoS one.

We shall look forward to hearing from you at your earliest convenience.

Journal Requirements:

When submitting your revision, we need you to address these additional requirements:

1. Please ensure that your manuscript meets PLOS ONE's style requirements, including those for file naming. The PLOS ONE style templates can be found at http://www.plosone.org/attachments/PLOSOne_formatting_sample_main_body.pdf and <http://www.plosone.org/attachments/PLOSOne_formatting_sample_title_authors_affiliations.pdf>

: We have edited our manuscript to meet PLOS ONE’s style requirements as the PLOS ONE style templates

2. In ethics statement in the manuscript and in the online submission form, please provide additional information about the patient records/samples used in your retrospective study. Specifically, please ensure that you have discussed whether all data/samples were fully anonymized before you accessed them and/or whether the IRB or ethics committee waived the requirement for informed consent. If patients provided informed written consent to have data/samples from their medical records used in research, please include this information.

: Thank you for your comment. All data were fully anonymized before we accessed them and this study was retrospective study therefore, our IRB committee waived the requirement for informed consent. And we added this sentence at method part in manuscript.

3. Thank you for including your ethics statement:

This retrospective cohort study adhered to the tenets of the Declaration of Helsinki and was approved by the Institutional Review Board (UUH 2017-09-037).

Please amend your current ethics statement to include the full name of the ethics committee/institutional review board(s) that approved your specific study.

: We amended our current ethics statement (UUH 2017-09-037-001) and included full name of the ethics committee to revised manuscript.

Once you have amended this/these statement(s) in the Methods section of the manuscript, please add the same text to the “Ethics Statement” field of the submission form (via “Edit Submission”).

: We add the same text to the ethics statement filed of the submission form

For additional information about PLOS ONE ethical requirements for human subjects research, please refer to http://journals.plos.org/plosone/s/submission-guidelines#loc-human-subjects-research.

4. In your Data Availability statement, you have not specified where the minimal data set underlying the results described in your manuscript can be found. PLOS defines a study's minimal data set as the underlying data used to reach the conclusions drawn in the manuscript and any additional data required to replicate the reported study findings in their entirety. All PLOS journals require that the minimal data set be made fully available. For more information about our data policy, please see http://journals.plos.org/plosone/s/data-availability.

Upon re-submitting your revised manuscript, please upload your study’s minimal underlying data set as either Supporting Information files or to a stable, public repository and include the relevant URLs, DOIs, or accession numbers within your revised cover letter. For a list of acceptable repositories, please see http://journals.plos.org/plosone/s/data-availability#loc-recommended-repositories. Any potentially identifying patient information must be fully anonymized.

Important: If there are ethical or legal restrictions to sharing your data publicly, please explain these restrictions in detail. Please see our guidelines for more information on what we consider unacceptable restrictions to publicly sharing data: http://journals.plos.org/plosone/s/data-availability#loc-unacceptable-data-access-restrictions. Note that it is not acceptable for the authors to be the sole named individuals responsible for ensuring data access.

We will update your Data Availability statement to reflect the information you provide in your cover letter.

: Data are available from ulsan university hospital ethics committee (contact via 0716782@uuh.ulsan.kr/Ms. Jeong Sook Kim; secretary of ulsan university hospital institutional review board)

5. Your ethics statement must appear in the Methods section of your manuscript. If your ethics statement is written in any section besides the Methods, please move it to the Methods section and delete it from any other section. Please also ensure that your ethics statement is included in your manuscript, as the ethics section of your online submission will not be published alongside your manuscript.

: Our ethics statement was appeared in methods section as “This retrospective cohort study adhered to the tenets of the Declaration of Helsinki and was approved by the ulsan university hospital Institutional Review Board (UUH 2017-09-037-001). All subjects were enrolled between June 2017 and September 2018.”

[Note: HTML markup is below. Please do not edit.]

Reviewers' comments:

Reviewer's Responses to Questions

 Review Comments to the Author

Please use the space provided to explain your answers to the questions above. You may also include additional comments for the author, including concerns about dual publication, research ethics, or publication ethics. (Please upload your review as an attachment if it exceeds 20,000 characters)

Reviewer #1: Lines 43-44: Globally the prevalence of AMD is higher than RVO, please correct and cite.
: First of all, thank you for your precious time on reviewing our manuscript. As you mentioned, we corrected that sentence and we added right citation.

Lines 48 – 50. Authors should be careful in associating pathophysiology of BRVO with NTG. These two diseases could maybe be associated with similar signs evaluated with a routine fundus ophthalmoscopy, but the mechanism can be different. Authors should clarify that NTG is more often associated with status of hypotension, headache and generalized vasoconstriction of blood vessels especially on the extremities.

: Thanks for your kind and detail review. As you mentioned, this sentence could lead to misunderstanding to reader, therefore, we changed this sentence more clearly and specifically and we added above sentence which you recommended.“This suggests that BRVO and glaucoma, particularly normal-tension glaucoma (NTG), may have a similar pathological mechanism such as blood flow insufficiency, NTG is more often associated with status of hypotension, headache and generalized vasoconstriction of blood vessels especially on the extremities though”

Lines 89-91: Authors should mention OCT tomography as useful tool to support the diagnosis of BRVO detecting retinal thinning and PAMM lesions.

(En Face Optical Coherence Tomography Analysis to Assess the Spectrum of Perivenular Ischemia and Paracentral Acute Middle Maculopathy in Retinal Vein Occlusion.
Ghasemi Falavarjani K, Phasukkijwatana N, Freund KB, Cunningham ET Jr, Kalevar A, McDonald HR, Dolz-Marco R, Roberts PK, Tsui I, Rosen R, Jampol LM, Sadda SR, Sarraf D.
Am J Ophthalmol. 2017 May;177:131-138. doi: 10.1016/j.ajo.2017.02.015. Epub 2017 Feb 22)

: As you mentioned, we added OCT tomography as tool to support the diagnosis of BRVO and also add citation what you recommended.

Lines 113-114: Can you specify the manufacturer’s scale given it is different among different companies?

:Manufacturer have recommended that image quality value must be 30 or higher, And we wanted to get high quality image, So we selected images which’s image value were over 35

Lines 156-158: Authors should specify the thickness of the slab they used to segment the choriocapillaris.

:As you mentioned, we specified slab of choriocapillary segement as “ Slab of choriocapillaris was identified by imaging a 23-μm area posterior from the Bruch membrane by automated segmentation “

Lines 200-201: Limitation of this retrospective study is the different size of the two groups.

: We added this sentence at limitation of our manuscript as you mentioned

I would add a paragraph describing the details of the algorithm used to perform the analyses.

: We used similar analyzing method which previous researcher used with DRI OCT Triton, so we think that we fully described how to analyze the data. However, I added some detail contents required for analysis with DRI OCT in method section

Reference) 1. Tang FY et al. Determinants of quantitative optical coherence tomography angiography metrics in patients with diabetes. Sci Rep.2017 Mya 31;7(1)

2. Sun Z et al. OCT angiography metrics predict progression of diabetic retinopathy and development of diabetic macular edema: a prospective study. Ophthalmology.2019 Dec ; 126

Was the peripapillary atrophy taken into consideration? Was it excluded from the analyses? Differences can also be affected by different extension of the peripapillary atrophy in the two groups so I would repeat the analyses and correct for that factor.

: We already considered peripapillary atrophy (PPA), especially beta- zone before analyzing of this study. Therefore, we excluded high myopia to avoid including PPA cases, because PPA is frequently related with myopia, especially high myopia. Fortunately, mean of spherical equivalent of both group(BRVO, NTG) were not severe ( -1.25 diopter, -2.45 diopter respectively). Moreover, in case of PPA presentation, we manually controlled circle for optic nerve circumference to include PPA also, not to analyze PPA to peripapillary capillaries before we analyzed data.

Reviewer #2: Major Concern:
Method of estimating capillary density
In this study capillary density is being measured using an average pixel intensity, which is a very crude measurement and could be affected by many confounding factors (for example, image contrast and levels due to slightly different quality of images could have a significant effect). How does exclusion of large vessels (briefly noted in lines 182:183) affect these measurements? Simple removal of large vessels (replacing those pixels with black) would have a significant impact on the average pixel intensity. Moreover, based on the description of methods in lines 167:170, the area of retina being analyzed will be different for each subject based on different Optic Nerve (ON) circle sizes. How was this controlled for? Is there a difference in ON size between NTG group and the BRVO group? In a normal eye does the density of peripapillary capillaries/choriocapillaris changes with distance from ON?

None the sources cited (references 23-36) validate this method for estimation of capillary density, let alone retinal capillary density with OCT. There are numerous Ophthalmology OCT-A studies that look at capillary density that have developed more accurate methods for measuring capillary density, and one of these validated techniques should be used in this paper. For examples of established methods see:

Geyman LS, Garg RA, Suwan Y, et al. Peripapillary perfused capillary density in primary open-angle glaucoma across disease stage: an optical coherence tomography angiography study. British Journal of Ophthalmology. 2017;101:1261-1268.

Fard et al. (2018) Pattern of peripapillary capillary density loss in ischemic optic neuropathy compared to that in primary open-angle glaucoma. PLoS ONE 13(1): e0189237. https://doi.org/10.1371/journal.pone.0189237

Mansoori et al. Radial Peripapillary Capillary Density Measurement Using Optical Coherence Tomography Angiography in Early Glaucoma. J of Glaucoma. 2017; 26(5):438-443. doi: 10.1097/IJG.0000000000000649

Mansoori et al. Measurement of Radial Peripapillary Capillary Density in the Normal Human Retina Using Optical Coherence Tomography Angiography. J Glaucoma. 2017 Mar;26(3):241-246. doi: 10.1097/IJG.0000000000000594.

Florence Coscas, Alexandre Sellam, Agnès Glacet-Bernard, Camille Jung, Mathilde Goudot, Alexandra Miere, Eric H. Souied; Normative Data for Vascular Density in Superficial and Deep Capillary Plexuses of Healthy Adults Assessed by Optical Coherence Tomography Angiography. Invest. Ophthalmol. Vis. Sci. 2016;57(9):OCT211-OCT223. doi: https://doi.org/10.1167/iovs.15-18793.

Jorge S. Andrade Romo, Rachel E. Linderman, Alexander Pinhas, Joseph Carroll, Richard B. Rosen, Toco Y. P. Chui; Novel Development of Parafoveal Capillary Density Deviation Mapping using an Age-Group and Eccentricity Matched Normative OCT Angiography Database. Trans. Vis. Sci. Tech. 2019;8(3):1. doi: https://doi.org/10.1167/tvst.8.3.1.

: Thanks for your valuable critiques and providing novel citation. However, in citation what you showed, they used different SS-OCT, Avanti OCT. It meant that built in program to show image was totally different with DRI –OCT. Moreover, image analyzing program such as SSAD, MATLAB could not be used to our study. Therefore, we looked for best way which extracted image and anazlyzed image with minimally compromising data. So, we took idea from quotation which we citied in manuscript. And our method is similar with tang ‘s way ^1,2^, because they used DRI-OCT instead using Avanti-OCT. For example, we used the latest built-in software (IMAGEnet6) to generated OCT-angiograms which can provide improved detection sensitivity of low blood flow and reduced motion artifacts without compromising axial resolution.^3^ Moreover, we tried to do our best to reduced confounding factors which can compromise data. For example, In cases of PPA, we excluded high myopia to avoid including PPA cases, because PPA is frequently related with myopia, especially high myopia. Fortunately, mean of spherical equivalent of both group(BRVO, NTG) were not severe ( -1.25 diopter, -2.45 diopter respectively). Moreover, in case of PPA presentation, we manually controlled circle for optic nerve circumference to include PPA also, not to analyze PPA to peripapillary capillaries before we analyzed data. With same context, there were no significant difference of ON size between both group. And when we excluded large vessels manually with i-solution program, there was no changing with pixel average. Also we did check intraclass correlation coefficient and Within-visit repeatability for reducing data compromising. Finally, before analyzing data with i-solution, we already noticed difference of OCTA between both group by checking manually, so it was nature and clear that same results was showed after analyzing with analyzing program, because analyzing program was one of artificial program just to detect factors which was shown in real life.

Reference) 1. Tang FY et al. Determinants of quantitative optical coherence tomography angiography metrics in patients with diabetes. Sci Rep.2017 Mya 31;7(1)

2. Sun Z et al. OCT angiography metrics predict progression of diabetic retinopathy and development of diabetic macular edema: a prospective study. Ophthalmology.2019 Dec ; 126

3. Stanga, P.E et al. Swept-source optical coherence tomography angio(Topcon Corp.Japan): Technology Review. Dev ophthalmol 56,13-17(2016)

Other comments:

Line 48-50:
The statement “This suggests that BRVO and glaucoma, particularly normal-tension glaucoma (NTG), share a common pathological mechanism” is not supported by the provided citations.

The cited studies show an association between elevated IOP/increase CD ratio and retinal vein occlusion (RVO), but do not imply that BRVO and glaucoma have same pathological mechanism. They suggests an increased risk of BRVO with glaucoma caused by changes in the retina that occur due to the glaucoma or directly due to elevated IOP (cause and effect, rather than a common underlying etiology). Furthermore, these studies suggest a link between RVO and elevated IOP, not normal tension glaucoma.

If you are indeed suggesting that BRVO and NTG share a common pathological mechanism please provide more evidence. Also please provide a more in depth explanation as to what the common underlying mechanism is, and why you are focused on NTG rather than glaucoma with elevated IOP

: Thanks for your kind and detail review. As you mentioned , this sentence could lead to misunderstanding to reader, therefore, we changed this sentence more clearly and specifically and we we added above sentence which you recommended.“ This suggests that BRVO and glaucoma, particularly normal-tension glaucoma (NTG), have a similar pathological mechanism such as blood flow insufficiency, NTG is more often associated with status of hypotension, headache and generalized vasoconstriction of blood vessels especially on the extremities though “

Line 67:
Suggest avoiding using “accordingly” to start two paragraphs in a row; It can simply be deleted here.
: As you mentioned, we deleted “accordingly”

Methods Questions:
For the 43 patients with NTG, which eye was selected and why? Please elaborate.
: We selected eye of NTG by following criteria 1) eye of unilateral NTG patients 2) In case of bilateral NTG patients, we selected eye with higher resolution OCT image 3) In case of similar resolution of OCT image with bilateral NTG patients, we selected right eye to minimalize selection bias. We add this sentence to method part in our manuscript.

Line 105-106:
This statement is poorly worded, and seems to state that the depth the OCT can scan is only 8um in depth, which is clearly not true. It is the In-depth resolution of the device that is 8um. Please clarify.
: As you mentioned , we changed as a axial resolution of 8 μm

Line 107:
Suggest changing this from “constant signal strength of the posterior pole” to constant signal strength throughout the posterior pole”
: As you mentioned , we changed to “constant signal strength throughout the posterior pole”

Line 153:
“Hypoechoic lesion” is the wrong term. Hypo/hyperechoic refer to ultrasound, not OCT which uses light rather than sound. Please use the correct terms (hyper/hyporeflective). Additionally, the inner plexiform layer is not a lesion, it should be referred to as a band. Please correct
: We totally agree with your recommendation, so, we corrected from hypoechoic to hyporeflective and we deleted layer from inner plexiform layer.

TABLE 1:
No need to include both % and (n) for sex, as one can easily be calculated from the other. Recommend consistent reporting of data, use the same format that you choose for reporting OD/OS.
: As you recommend, we deleted % of sex to show same format that we chose for reporting OD/OS.

Line 218-219:
Please include the units of ALCD mean difference
: As you recommend, we include the units of ALCD mean difference as μm

Line 217 and Table 4:
ALCD vs ALDc Do these acronyms represent the same thing? If so, why use two acronyms? Please clarify the difference. Also suggest using a different acronym to distinguish mean ALDc from ALDc used in Table 2
: AS you commended , this terms could be confused . ALCD was anterior laminal cribrosa depth and mean difference of ALCD was calculated values of mid-superior level of anterior lamina depth of central side (ALDc) minus values of mid-inferior level of anterior lamina depth of central side(ALDc). So, we wanted to emphasize that we used anterior lamina depth of central side (ALDc). However, as you mentioned, it may be confused, so, we changed ALDc in table to ALCD because using ALDc was described at title of Table.

Line 268-270:
You state “… LTC can decrease in cases with ganglion cell degeneration and axonal damage, with consequent inhibition of axonal transport.”
Are you saying inhibition of axonal transport a consequence of RGC degeneration or is it a cause? This is a controversial topic and this statement should be re-worded or supported by citations.
: Quigley et al. said that the actual cause of early optic nerve head cupping in glaucoma appears to be the loss of axonal tissue in their studies. So, we added this studies as citations.

Line 270-271
The analogy comparing LC weakening to a concrete building is very clever. I have never heard it before, but it is great way to visualize this.
: Thanks for your comment

Line 281-282
An ischemic lesion would not cause “focal axonal transection,” which implies cutting. Please reword this.

 : In Alshareef’s study( reference 37) ,they also expressed similar sentence , however it may cause misunderstanding , so we changed transection to dysfunction

 Thank you for your valuable critiques and positive comments on our manuscript. We hope that the revisions made according to your comments may fulfill the requirements for publication in PLOS one.
