# Supplementary material for: Comparison of lamina cribrosa properties and the peripapillary vessel density between branch retinal vein occlusion and normal-tension glaucoma
Source: PLoS One. 2020 Oct 2;15(10):e0240109. doi: 10.1371/journal.pone.0240109 (PMC7531791; doi:10.1371/journal.pone.0240109)
Supplement: Attachment — Submitted filename: Response to Reviewer.docx [file pone.0240109.s002.docx]

Dear Editor:

We wish to re-submit the attached manuscript as an Original Article. The manuscript ID is PONE-D-20-01336R1.

The manuscript has been rechecked and appropriate changes have been made in accordance with the reviewers’ suggestions. The responses to their comments have been prepared and attached given below.

We thank you and the reviewers for your thoughtful suggestions and insights, which have enriched the manuscript and produced a better and more balanced account of the research. We hope that the revised manuscript is now suitable for publication in your journal.

Thank you for your consideration. I look forward to hearing from you.

Sincerely,

Chang Kyu Lee, MD

Department of Ophthalmology

Ulsan University Hospital

University of Ulsan College of Medicine

Ulsan, South Korea

coolleo@uuh.ulsan.kr

Reviewer #1: Thank you for taking the time revising your manuscript and understanding our queries.

I only have one further query on the choriocapillaris segmentation. We all know that OCTA gives us the unprecedented advantage to quantify choriocapillaris, yet there are many limitations related to its use. In order to obtain the most reliable results we have to control for many confounders. Authors stated that they used a default slab 23-um thick posteriorly to the Bruch using default segmentation.
: Thank you for your pertinent query. As you know, the choroid is divided into five layers. Starting from the retinal side, these include Bruch’s membrane, three vascular layers (the choriocapillaries; Sattler’s layer, which is composed of medium and small arterioles; and Haller’s layer, which includes large arteries and veins), and the suprachoroidea. Torczynski et al. found mean thickness of choriocapillaries was 27.5 μm by histological section after autopsy, and recently, Uji et al. showed the mean diameter of choriocapillary vessels was 22.8 μm by using OCTA. Moreover, choriocapillaries lie directly under Bruch’s membrane, while large vessels of the system lie posterior to the capillaries. We wanted to increase the proportion of choriocapillaries seen; therefore, we set the starting segmentation from under Bruch’s membrane, which is a hyperreflective band by OCTA and has no vascular area in a retinal lesion, and the ending segmentation was set to 23 μm below Bruch’s membrane. In every case, we checked whether the starting segmentation included Bruch’s membrane, or was over Bruch’s membrane not including the RPE layer. If there was such a case, then we manually corrected the starting segmentation not to include Bruch’s membrane or the area over it.

I have a few queries.

1. Automated segmentation is not enough to correct segmentation errors, manual correction is needed. Could please the Authors repeat the analysis correcting all the segmentation errors?
: Thank you for your concern. We provided an answer to this query in the above paragraph.

2. Why did the Authors choose a 23 um thick slab given histologically the CC is reported to be 10-um thick?
: We appreciate the question. We provided an answer to this in the above paragraph.

3. My other concern is the position of the CC slab. From my understanding the Authors are locating the slab right off the Bruch membrane, right? The selected slab is very close to the RPE and I assume many flow deficits are coming from RPE projection. Did the Authors confirmed the results using a deeper slab?

: We further described our methods in the above paragraph.

Reference)

1. [Joanna Kur](http://pubmed.ncbi.nlm.nih.gov.ssl.libproxy.amc.seoul.kr:8000/?term=Kur+J&cauthor_id=22580107) [^1^](http://pubmed.ncbi.nlm.nih.gov.ssl.libproxy.amc.seoul.kr:8000/22580107/#affiliation-1), [Eric A Newman](http://pubmed.ncbi.nlm.nih.gov.ssl.libproxy.amc.seoul.kr:8000/?term=Newman+EA&cauthor_id=22580107), [Tailoi Chan-Ling](http://pubmed.ncbi.nlm.nih.gov.ssl.libproxy.amc.seoul.kr:8000/?term=Chan-Ling+T&cauthor_id=22580107) Cellular and Physiological Mechanisms Underlying Blood Flow Regulation in the Retina and Choroid in Health and Disease. Prog Retin Eye Res 2012 Sep;31(5):377-406
2. [E Torczynski](http://pubmed.ncbi.nlm.nih.gov.ssl.libproxy.amc.seoul.kr:8000/?term=Torczynski+E&cauthor_id=1266921), [M O Tso](http://pubmed.ncbi.nlm.nih.gov.ssl.libproxy.amc.seoul.kr:8000/?term=Tso+MO&cauthor_id=1266921). The Architecture of the Choriocapillaris at the Posterior Pole. Am J Ophthalmol 1976 Apr;81(4):428-40
3. Reference 22 in manuscript

Reviewer #2: My major concern regarding the method of estimating capillary density was not adequately addressed by the authors response. The author provided several addition sources, however none of these sources justify using a crude average pixel intensity to estimate capillary density. All of the provided sources use image analysis programs to identify vessels and calculate capillary density in a much more reliable way. While many of these sources take multiple images, align them, and average the aligned images together in order to decrease the signal to noise ratio and aid in vessel identification, none of them use average pixel intensity as an estimate of capillary density. There are too many confounding factors that could affect the average intensity of a single image/ROI. While I appreciate that resources may limit the authors access to some of the software or custom analysis tools used by other groups, this type of analysis can be done using free software such as ImageJ.
Furthermore, the figure in which this data is shown (Fig 4) is very unclear. Average pixel intensity is displayed as a percent, but a percent of what? It is also unclear what the error bars represent (SEM vs SD), and there is significant overlap of error bars for all 12 comparisons. Additionally, the methods state that independent t-tests were used for all statistical analysis, however this type of analysis is inappropriate for this data. This data is comparing multiple dependent variables between two independent groups (ie capillary density of the superior nasal region and capillary density of the inferior nasal region in a single patient are dependent, while the NTG and BRVO groups are independent). Independent t-tests are commonly misused in the analysis of this type of data, and can lead to type I errors especially as the number of dependent variables increases (in this instance there are 12 dependent variables increasing the likelihood of type 1 error). A more appropriate analysis for this data would be one-way multivariate analysis of variance.
Please see the following article on the misuse of t-tests in medical research:
Guangping Liang, Wenlaing Fu, and Kaifa Wang. Analysis of t-test misuses and SPSS operations in medical research papers. Burns Trama. 2019; 7:31.
: We sincerely regret that our previous response did not fully address your major concern.

First, we must explain i-solution software. i-solution is image analysis software that is similar to image J. However, I believe it is more convenient compared to image J, because it is a well-designed platform to analyze images. i-solution software has been used in the basic research field for detection of cell number, counting axons of neurons, and in the heavy material field to calculate exact length and exact area from acquired images. Uses of i-solution in basic research were cited in the manuscript.

Moreover, our procedure of image analysis was very similar to that of the Mansoori study, which was provided previously. To summarize processing, there are 3 main stages. The first stage is extraction of the region of interest (ROI) around the ONH. The second stage is detection and suppression of thick vessels. In this stage, there was some difference between our study and that of Mansoori. They used the Bar-Selectiye combination of shifted filter responses method, but in our study, we excluded large vessels such as the superior and inferior retinal artery and vein one-by-one using sub mode (deletion), a built-in program of i-solution. Even though there was some worry about not fully suppressing large vessels using this process, in the newly provided image (Figure 3) we can see that almost all large vessels were excluded. The last stage is estimation of peripapillary vessel density, which is the ideal measure of capillaries per unit area. The desired image is the binary image, in which white pixels represent capillaries (without large vessels) and black pixels represent non-vessels. The peripapillary vessel density was calculated using the following formula: peripapillary vessel density = (N_w_ / A )× 100, where N_w,_ represents the number of white pixels and A represents the area of the selected image sector. As both the numerator and the denominator are pixel counts, the peripapillary vessel density is reported between 0% and 100%. We included these processes and the figures in the methods section of the manuscript.

The error bar in Figure 4 represents standard deviation (SD), and we included these contents in the Figure 4 legend.

We purposefully discussed the statistical analysis of Figure 4 with the statistician in the medical information and research center of our hospital. We finally concluded that your suggestion was more reasonable, and we rechecked our data with one-way multivariate analysis of variance (MANOVA). After this, there were no significant changes in p-values, and we have included the results of MANOVA below. So, we needed not change Figure 4, and we concluded using figure 4 continuously instead of table, because in the figure we easily see the differences at a glance.

Other Minor Points:
Line 43: Sentence is grammatically incorrect. Should read “Retinal vein occlusion is a common cause of vision loss…” or “Retinal vein occlusion is one of the most common causes of vision loss…”
: We changed this sentence as you recommended and double-checked it using Editage.

Line 76: Need to capitalize Ulsan University Hospital
: We changed this sentence as you recommended and double-checked it using Editage.

Line 161: should read “…at the inner plexiform layer, which is the most superficial hyporeflective band.”

: We changed this sentence as you recommended and double-checked it using Editage.
