# Supplementary material for: Comparison of lamina cribrosa properties and the peripapillary vessel density between branch retinal vein occlusion and normal-tension glaucoma
Source: PLoS One. 2020 Oct 2;15(10):e0240109. doi: 10.1371/journal.pone.0240109 (PMC7531791; doi:10.1371/journal.pone.0240109)
Supplement: Attachment — Submitted filename: Response to Reviewer.docx [file pone.0240109.s003.docx]

Reviewer #1: Thank you for the time spent revising my comments.

I have a main concern related to the choriocapillaris quantification.

The Authors mentioned that as published recently, Uji et al showed the mean diameter of choriocapillary vessels was 22.8 μm by using OCTA. These findings are simply describing the mean diameter of the vessels, not referring to the location of CC and thickness.

More recently, Byon et al published on the most repeatible and reliable CC slab in quantitative studies. I would suggest to revise the following manuscript and consider improving the methodology.


Byon I, Alagorie AR, Ji Y, Su L, Sadda SR. Optimizing the Repeatability of Choriocapillaris Flow Deficit Measurement from Optical Coherence Tomography Angiography [published online ahead of print, 2020 May 23]. Am J Ophthalmol. 2020;S0002-9394(20)30266-X. doi:10.1016/j.ajo.2020.05.027

:Thank you for your pertinent suggestion. Accordingly, we rechecked the choriocapillaris with the method used by Byon et al, that is,a slab of choriocapillaris was identified as a 10-μm thick slab offset of 21μm below the instrument generating the retinal pigment epithelial band and have added this information in the manuscript. There was no significant difference between the results obtained by the new method and that obtained by the previously used result.

We think that one of the reasons for the above result was that we manually corrected the starting segmentation to not include the Bruch’s membrane or the area over as was done in our previous interpretation.

We have presented this new result as Figure 4.

Reviewer #2: Thank you for further clarifying your analysis of vessel density. Prior descriptions of the analysis methods did not make it clear that images were converted from gray scale to a binary image. Given this clarification, I agree that the method of analysis used here is appropriate. My only suggestion would be to include an example of the final binary image in figure 3, as the images shown in are still in gray scale. Additionally, the authors re-analyzed this data using one-way multivariate analysis of variance and addressed my concerns regarding appropriate statistical analysis.

:Thank you for your comment. Accordingly, we have added the final binary image in Figure 3 as part D.
